# A Treatise on Hydrocephalus, or Water on the Brain; with the Most Successful Modes of Treatment

**Published:** 1836-04

**Authors:** 


Art. XVI.—*A Treatise on Hydrocephalus, or Water on the Brain; with the most successful modes of Treatment.* By William Griffith, Lecturer on Midwifery and the Diseases of Women and Children, at the Westminster School of Medicine, &c. *London*, 1835. 12mo. pp. 86.

We are compelled by an imperious sense of duty, although with unfeigned reluctance, to speak of this work in terms of disapprobation. We are altogether unacquainted with the gentleman who is the author of it, and we may truly, and certainly without any disparagement, say that even his name was unknown to us, until we saw it on his title-page. And yet, we may be permitted to add, as well for ourselves as in behalf of critics generally, that if his work meets with a different reception from that which he could desire, he has to thank himself alone for this; inasmuch as its publication is an act purely gratuitous on his part. The subject of which it treats is familiar to every practitioner, although certainly much yet remains to be done, both for its pathology and treatment; it contains not, nor does it profess to contain, one particle of novelty in doctrine or practice; there exist numerous treatises on the same disease much fuller and more accurate, and accessible to every reader; and assuredly, the work displays no indication of that irrepressible spring of genius, that "divine thrusting-on," which often obliges the young enthusiast to make a premature display of his powers, ere yet chastened by experience.

The faults of this work are indeed so numerous, its merits so insignificant, that we do not doubt but that its publication will hereafter be a subject of regret to its author, when maturer experience and a cooler judgment (for we presume, although we know it not, that Mr. Griffith is still young,) have led him to form a juster estimate of what is required of those who step forth from the privacy of life as professed guides of their brethren in a difficult route.

Compared with many—we had almost said with all previous treatises on hydrocephalus with which we are acquainted in English, French, or German, we must say that Mr. Griffith's is in the highest degree meager in the enunciation of well ascertained facts relative to the disease; it is full of tame truisms, gratuitous assumptions, false pathology, and illogical inferences; and even its practical precepts are by no means always judicious. In regard to its literary character, we must say that its plan is confused, that its materials are ill-arranged, and that its language is far from accurate.

And yet we are gratified to be able to say, in parting with Mr. Griffith, that we here and there perceive in his little work the indications of a talent for observation and practical application which probably renders him a skilful practitioner, and may well entitle him to the confidence of his friends. The faults of his book are also, perhaps, as much the consequence of that contagious rage for premature publication, for which the members of our profession have been for some years past unhappily distinguished, and of that careless and vicious style both of thinking and writing, which the false lenity of medical criticism has tended to foster, as of any defect either of talent or practical experience in the author. And we shall be far from surprised, if his next appearance before the public should claim for us a meed of approbation as sincere, and far more cordial than the censure which the overmastering sense of public duty has now wrung from us, reluctantly and painfully.

